# Photobiomodulation at 660 nm Stimulates In Vitro Diabetic Wound Healing via the Ras/MAPK Pathway

**DOI:** 10.3390/cells12071080

**Published:** 2023-04-04

**Authors:** Patricia Kasowanjete, Heidi Abrahamse, Nicolette N. Houreld

**Affiliations:** Laser Research Centre, University of Johannesburg, Johannesburg 2006, South Africa; jkasowanjete@gmail.com (P.K.); habrahamse@uj.ac.za (H.A.)

**Keywords:** diabetes, MAPK, signal transduction, photobiomodulation, wound healing, Ras

## Abstract

Diabetic foot ulcers (DFUs) are open chronic wounds that affect diabetic patients due to hyperglycaemia. DFUs are known for their poor response to treatment and frequently require amputation, which may result in premature death. The present study evaluated the effect of photobiomodulation (PBM) at 660 nm on wound healing via activation of Ras/MAPK signalling in diabetic wounded cells in vitro. This study used four human skin fibroblast cell (WS1) models, namely normal (N), wounded (W), diabetic (D), and diabetic wounded (DW). Cells were irradiated at 660 nm with 5 J/cm^2^. Non-irradiated cells (0 J/cm^2^) served as controls. Cells were incubated for 24 and 48 h post-irradiation, and the effect of PBM on cellular morphology and migration rate, viability, and proliferation was assessed. Basic fibroblast growth factor (bFGF), its phosphorylated (activated) receptor FGFR, and phosphorylated target proteins (Ras, MEK1/2 and MAPK) were determined by enzyme-linked immunosorbent assay (ELISA) and Western blotting; nuclear translocation of p-MAPK was determined by immunofluorescence. PBM resulted in an increase in bFGF and a subsequent increase in FGFR activation. There was also an increase in downstream proteins, p-Ras, p-MEK1/2 and p-MAPK. PBM at 660 nm led to increased viability, proliferation, and migration as a result of increased bFGF and subsequent activation of the Ras/MAPK signalling pathway. Therefore, this study can conclude that PBM at 660 nm stimulates in vitro diabetic wound healing via the bFGF-activated Ras/MAPK pathway.

## 1. Introduction

Diabetes Mellitus (DM) is a chronic metabolic condition characterised by a lack of insulin, the absence of insulin, or resistance to insulin. DM remains a critical global health concern affecting different ages of the population. The current statistics indicate that South Africa has a prevalence of 12.7% in adults aged 20–79 years [1]. Diabetic patients suffer from delayed and non-healing diabetic wounds such as diabetic foot ulcers (DFUs). Chronic diabetic wounds are known for their non responsiveness to treatment and frequently require amputation, and at times result in premature death [2]. Despite the effort to control the disease and improve healing, available current therapies have only achieved a 50% healing rate, indicating that very little progress has been made in impeding diabetic wounds [3]. Diabetic patients are impacted physically, mentally, and financially as a result of these chronic non-healing wounds, as well as their families, governments, and health care systems due to the substantial financial impact placed on such individuals and agencies in terms of treatment [2].

The delay in wound healing is associated with compromised cellular processes, which results in reduced cellular migration and proliferation, as well as poor production of growth factors and collagen synthesis [4]. Wound healing involves a series of molecular and cellular activities that target the repair of damaged tissue. Growth factors, including fibroblast growth factor (FGF), are the main players in the regulation and redirection of the process of wound healing [5]. It is widely known that diabetic wounds lack several growth factors due to their reduced production and faster rate of destruction [6], and several signalling pathways are dysregulated. FGF plays an important role in wound healing, including cell growth, regulation, division, differentiation, and cell migration. The binding of FGF to its corresponding signalling receptor (FGFR) activates the receptor in the presence of heparan sulphate (HS) as a co-factor, leading to the formation of an FGF–FGFR–HS complex. The intracellular tyrosine kinase domain of FGFR is phosphorylated or activated, leading to the stimulation of downstream signalling pathways. FGFR is linked to various intracellular signalling pathways, including the Ras/MAPK pathway, which plays a critical role in wound healing [5]. When FGFR gets activated (phosphorylated), the signal is transmitted to Ras, Raf and MEK, which, in turn, activates MAPK, which then translocates to the nucleus, where it activates genes involved in cellular proliferation, migration, differentiation, and angiogenesis [7]. Ras/MAPK is the most studied signalling pathway, and its primary function is to relay signals from outside the cell to the nucleus, where specific genes are activated for cellular division, growth and survival, motility, and differentiation [5,7,8]. Therapies which can target this signalling pathway to stimulate wound healing would be advantageous.

Photobiomodulation (PBM) has been utilised to cause physiological changes and provide therapeutic effects. PBM stimulates biological processes in an organism using the light of a specific wavelength [9]. The absorbed light promotes and activates numerous cellular processes, resulting in downstream physiological effects, such as improved wound healing and reduced inflammation, oedema, and pain. PBM has been used to treat numerous conditions and has been shown to be effective in the treatment of chronic wounds [4,10]. PBM using red light is frequently used to treat chronic wounds either alone or in combination with near infrared (NIR) wavelengths. PBM has been shown to enhance cellular viability and proliferation as well as the release of growth factors such as FGF, epidermal growth factor (EGF), vascular endothelial growth factor (VEGF), platelet-derived growth factor (PDGF), and insulin-like growth factor (IGF) [11,12]. These growth factors play an important role in the redirection and regulation of the process of wound healing. A similar recent study conducted by our research group showed that PBM at 660 nm and 5 J/cm^2^ altered cellular autocrine signalling, particularly the EGF/EGFR loop that activated the JAK/STAT pathway, which, in turn, promoted cell proliferation and migration in human fibroblast diabetic wounded cells in vitro [13].

Despite the overwhelming evidence supporting PBM’s therapeutic potential, PBM is still not widely accepted in the medical field as the effects it induces at the molecular, cellular, and tissue level have not been fully investigated. The influence of PBM in the visible red range on the FGF mediated Ras/MAPK pathway is not known. The aim of this present study was to investigate if PBM at 660 nm stimulates in vitro diabetic wound healing via the Ras/MAPK pathway.

## 2. Materials and Methods

### 2.1. Cell Culture

This research work was approved by the Faculty of Health Sciences Research Ethics Committee (1 June 2020, REC-487-2020) at the University of Johannesburg. Commercially available human skin fibroblast cells (WS1; ATCC^®^, CRL 1502™, Manassas, VA, USA) were used in this study. Cells were cultured in minimum essential media (MEM; M7278 Sigma-Aldrich, Johannesburg, South Africa) supplemented with foetal bovine serum (10%; FBS, F9665 Sigma-Aldrich, Johannesburg, South Africa), L-glutamine (2 mM; D6429 Sigma-Aldrich, Johannesburg, South Africa), 1% penicillin (10,000 units)-streptomycin (10 mg/mL; P42942 Sigma-Aldrich, Johannesburg, South Africa), 1% amphotericin B (250 µg/mL; A2942 Sigma-Aldrich, Johannesburg, South Africa), non-essential amino acid (0.1 mM; NEAA, 11140-035 Sigma-Aldrich, Johannesburg, South Africa), and sodium pyruvate (1 mM; S8636 Sigma-Aldrich, Johannesburg, South Africa). This study made use of four cell models, namely normal (N), wounded (W), diabetic (D), and diabetic wounded (DW).

A diabetic cell model was created by continuously growing the cells in media (already containing 5.6 mM glucose) containing an additional 17 mM D–glucose; thus, diabetic cells were grown in a total glucose concentration of 22.6 mM through several (continuous) cell passages [12]. Cells (6 × 10^5^) were seeded into 3.4 cm diameter culture plates and allowed to attach for 24 h. Thirty minutes prior to PBM, a ‘wound’ was created by the central scratch assay in all the wounded models (W and DW). The central scratch assay is a valuable tool for understanding the effect of experimental assays for promoting wound healing [14]. This assay is cheap and simple and is an invaluable method for understanding cell migration, favouring the quantification of cell migration under controlled experimental conditions [15]. This was created on the cell monolayer using a sterile 1 mL pipette and incubated for 30 min to allow the cells to adapt [12].

### 2.2. Laser Irradiation

The cells received irradiation using a continuous wave diode laser at a wavelength of 660 nm with a fluence of 5 J/cm^2^. The diode laser used in this study was provided and set up by the Council for Scientific and Industrial Research (CSIR)/National Laser Centre (NLC) (Pretoria, South Africa). A fluence of 5 J/cm^2^ was used in this study as it has been extensively used by our research group in vitro and shown to be an optimal fluence at a wavelength of 660 nm [13,16]. Non-irradiated cells acted as controls (0 J/cm^2^). The cells were irradiated in 3.4 cm diameter culture plates containing 1 mL of complete media (with additional D-glucose in the case of diabetic cells) from above via fibre optics in the dark at room temperature. The laser parameters used in this study are shown in Table 1. Thereafter, the target proteins were analysed at 24 and 48 h post-irradiation.

### 2.3. Cellular Morphology and Migration Rate

Cellular morphology and migration rate was assessed by inverted light microscopy (Olympus CKX41 and cell Sens imaging software version 2.3, Wirsam Scientific, Johannesburg, South Africa). To determine and calculate the migration rate in W and DW models, the distance between the wound margins in three places along the scratch (the average of which was used) was measured using the imaging software in images captured at 0, 24 and 48 h and used in the equation below,

(At_oh_ − At_time_)/At_oh_ × 100

where At_oh_ is the scratch area at 0 h and At_time_ is the correspondent scratch area at 24 or 48 h [17,18].

### 2.4. Cellular Viability

The Trypan blue exclusion assay was used to count the cells and to determine cellular viability. Trypan blue is a stain used to determine the number of live/viable cells, and its principle is based on dye exclusion; dead cells take up the dye due to their permeable cell membrane, while viable cells do not take up the dye because their cell membranes are impermeable. An equal amount of dye (10 μL; T8154, Sigma-Aldrich, Johannesburg, South Africa) and cells were mixed and loaded into a disposable Countess^®^ cell counting chamber slide. Unstained and stained cells were counted, and the viability percentage was determined using the Invitrogen Countess^®^ II FL Automated Cell Counter.

### 2.5. Cellular Proliferation

The BD Pharmingen™ BrdU Flow Kit (559619/557891 BD Biosciences, the Scientific Group, Roodeport, South Africa) was used to determine cellular proliferation. 5-Bromo-2-deoxyuridine (BrdU) is an analogue of the DNA precursor thymidine and is integrated into newly produced DNA during the synthesis (S) phase of the cell cycle. 7-Amino-actinomycin D (7-AAD) is also included for DNA analysis. Briefly, 1 × 10^6^ cells were cultured in a 3.4 cm diameter tissue culture plate, irradiated, and incubated as previously described. After 24 or 48 h incubation, cells were detached and fixed with 100 μL Cytofix/Cytoperm™ buffer and incubated for 30 min on ice. Thereafter, cells were rinsed in 1 mL 1xBD perm/wash buffer and centrifuged at 300× *g* for 5 min, and then incubated with 100 μL BD Cytoperm permeabilisation buffer plus on ice for 10 min. Cells were rinsed in 1 mL 1× BD perm/wash buffer, centrifuged for 5 min at 300× *g*, and the deposit was resuspended in 100 μL BD Cytofix/Cytoperm™ buffer and incubated on ice for 5 min. The cells were again washed as before and then treated with 100 μL DNase (300 μg/mL in DPBST) to expose incorporated BrdU and incubated for 1 h at 37 °C. After washing, cells were stained with 50 μL of BD perm/wash buffer containing diluted, fluorescently labelled anti-BrdU and incubated for 20 min at room temperature. Thereafter, the cells were washed and stained for total DNA by resuspending them in 20 μL of 7-AAD and resuspended in 1 mL of staining buffer. Cells were analysed using the Becton Dickinson (BD) Accuri C6 flow cytometer (BD Biosciences, the Scientific Group, Roodeport, South Africa) at a rate of 400 events per second with a 350 uL sample limit. Gates were drawn around the cells using control cells in the forward scatter (FSC-A) and side scatter (SSC-A) plot during acquisition using unstained samples.

### 2.6. Basic Fibroblast Growth Factor (bFGF)

In this study, bFGF released into the culture medium was determined by sandwich solid-phase ELISA as per the manufacturer’s instructions (KHG0021, Invitrogen™ Human FGF basic ELISA Kit, ThermoFisher Scientific, Johannesburg, South Africa). To prepare the sample, culture media from control cell models and PBM cell models was diluted 2-fold in standard diluent buffer. An 8-point standard curve (1000, 500, 250, 125, 62.5, 31.2, 15.6 and 0 pg/mL) was prepared and run with each plate. Sample and standard were added to the test 96-well plate (100 µL) in duplicate, the average of which was used. Plates were covered and incubated at room temperature for 2 h, after which they were washed three times. This was followed by the addition of 100 μL of Hu bFGF biotin conjugate and incubation at room temperature for 1 h. Plates were washed, followed by the addition of 100 μL 1× Streptavidin-horseradish peroxidase (HRP) and incubated at room temperature for 30 min. Following the washing step, 100 μL 3,3′,5,5′-tetramethylbenzidine (TMB) was added, and plates were incubated for 30 min at room temperature in the dark. Stop solution (100 μL) was added and the colour change reaction was measured spectrophotometrically at a wavelength of 450 nm using Victor Nivo^®^ multimode plate reader (Perkin-Elmer, Midrand, South Africa).

### 2.7. Phosphorylated Fibroblast Growth Factor Receptor (p-FGFR)

In this study, the Western blot assay was used to analyse p-FGFR. In order to prepare the sample, cells cultured in 3.4 cm diameter culture plates were washed three times using ice-cold phosphate-buffered saline (PBS). Adherent cells were removed using a cell scraper, transferred, and centrifuged for 5 min at 595× *g*. Thereafter, the supernatant was discarded, and the cells were resuspended in 200 μL ice-cold cell lysis buffer (9803, Cell Signalling Technology^®^, Anatech Instruments (Pty) Ltd., Johannesburg, South Africa). Twenty microliters of 1 mM fresh Phenylmethylsulphonyl (PMSF) protease (P-7626, Sigma-Aldrich, Johannesburg, South Africa) was added to 180 μL lysis buffer and incubated for 30 min on ice, and centrifuged at 13,709× *g* at 4 °C for 10 min. The protein mix (supernatant) was stored on ice and the protein concentration quantified using the Pierce™ BCA Protein Assay kit (23225, ThermoFisher Scientific, Johannesburg, South Africa). Laemmli 2× sample buffer (S3401, Sigma-Aldrich, Johannesburg, South Africa) was added to dilute the cell lysate at a loading protein concentration of 1 μg/μL and the mixture was then heated for 5 min at 95 °C.

Protein samples (20 μL) were separated via sodium dodecyl sulfate-polyacrylamide gel electrophoresis (SDS-PAGE) and transferred to an Immuno-Blot PVDF membrane (1620177, Bio-Rad, Sandton, South Africa) using a semi-dry blotter (B2529, Sigma-Aldrich, Johannesburg, South Africa) for 3 h with the power supply set at 60 volts.

A Millipore Sigma™ SNAP i.d™ 2.0 protein detection system was used for the immune detection within 30 min. Once the proteins were transferred, the PVDF membrane was blocked in 5% bovine serum albumin (BSA, in Tris-buffered saline, TBS) for 10 min. After the three cycles of washing with 0.1% Tween-20 in Tris-buffered saline (TBS), primary antibody was added (1:100 in BSA, phospho-FGFR1/FGFR2 (Tyro730, tyro733), polyclonal antibody, abx215351, Abcam, Biocom Africa, Pretoria, South Africa) and incubated for 1 h at room temperature. The membrane was then washed three times in 1% Tween-20 in PBS (10 min each) and incubated for 10 min at room temperature with horseradish peroxidase conjugate secondary antibody (1:1000 in PBST, Goat-anti-rabbit IgG (H&L); HAF2008, R&D Systems, Whitehead Scientific (Pty) Ltd. Modderfontein, South Africa) followed by washing three times in TBS.

The membrane was then incubated for 5 min in the dark in 1% 3,3′-Diaminobenzidine (DAB) and 0.3% hydrogen peroxide diluted in 5 mL PBS. Bands were scanned and photographed using a Bio-Rad ChemiDoc™ MP imaging system and Image Lab 5.2.1 software. GAPDH was used as loading control (sc-4774, Santa Cruz, Biotechnology, Anatech (Pty) Ltd., Johannesburg, South Africa). GAPDH (1:100 in BSA) was used as a loading control and incubated together with primary antibodies. ImageJ software (National Institute of Health, USA) was used to process and semi-quantitatively analyse the scanned densitometric estimates as ratios to the loading control.

### 2.8. Phosphorylated (p-) Ras

p-Ras was analysed using in-cell ELISA. Post-incubation cells were detached and 3 × 10^5^ cells were seeded into 96-well culture plates and allowed to attach in 200 μL media. After 4 h, 100 μL of culture media was removed and an equal amount of 8% paraformaldehyde was added to fix the cells for 15 min at room temperature. Thereafter, the plates were washed three times in 1x PBS-T (0.05% Tween-20 in PBS) and then incubated for 30 min at room temperature in 200 μL permeabilization buffer (1% Triton X-100 in PBS). This was followed by the addition of blocking solution (2% BSA, in PBS) and incubation at room temperature for 2 h. Thereafter, plates were rinsed three times with PBS-T, and 100 μL of 1:20,000 (diluted in 2% BSA) primary antibody was added (anti-phospho Ras-GRF1 (p-Ser915) SAB4504065, Sigma-Aldrich, Johannesburg, South Africa) and incubated at room temperature for 2 h. Plates were washed three times in wash buffer (0.05% Tween-20 in PBS) and 100 μL of 1:5000 biotin-labelled secondary antibody (Goat-anti-rabbit IgG, HRP conjugated, HF008, R&D Systems, Whitehead Scientific (Pty) Ltd. Modderfontein, South Africa) was added. Plates were incubated at room temperature for 2 h, washed three times, and TMB (100 uL) was added and incubated for 20 min at room temperature. Hydrochloric acid (1 M) was then added to stop the reaction and the colour change was measured at 450 nm using the Victor Nivo^®^ multimode plate reader (Perkin-Elmer, Midrand, South Africa).

### 2.9. Phosphorylated (p-) MEK1/2

The phospho-MEK (pSer217/221)/pan-Mek ELISA Kit (RAB0347, Sigma-Aldrich, Johannesburg, South Africa) was used for the measurement of p-MEK1/2 in human lysates. Cells were washed with PBS and solubilised in 250 μL 1× cell lysate buffer, and mixed and incubated with shaking for 30 min at 4 °C. The supernatant was used for analysis following centrifugation at 7571× *g* for 10 min at 4 °C. Samples were diluted 5-fold in sample dilution buffer, and 100 μL of sample/positive control was added into designated wells and plates incubated at room temperature for 2 h and 30 min of shaking. Plates were washed and incubated at room temperature with shaking for 1 h with 100 μL of 1× rabbit anti-phospho–MEK1/2. Plates were washed and incubated with 100 μL 1× HRP conjugated anti-rabbit IgG overnight with shaking at 4 °C. Plates were washed and 100 μL TMB was added, and plates were incubated in the dark at room temperature with shaking for 30 min, after which 50 μL stop solution was added. Absorbance was read at 450 nm using the Victor Nivo^®^ multimode plate reader (Perkin-Elmer, Midrand, South Africa).

### 2.10. Phosphorylated (p-) ERK1/2 (MAPK)

This study used the human p-ERK1/2 (phospho Extracellular Signal Regulated Kinase1/2) ELISA kit (E-EL-H1698, Elabscience^®^, Biocom Africa, Pretoria, South Africa) to analyse p-ERK1/2. Cells were rinsed with pre-cooled PBS, detached, and centrifuged for 5 min at 1000× *g*. The supernatant was discarded, and cells were washed three times with pre-cooled PBS. Thereafter, the cells were sonicated in 250 µL PBS (Branson 1800 1/2-gallon ultrasonic cleaner bath, 40 kHz). A 7-point stand curve (1000, 500, 250, 125, 62.5, 31.25 and 0 pg/mL) was included and 100 μL of standard/sample was added in duplicate and incubated at 37 °C for 90 min. Following incubation, plates were washed and 100 μL biotinylated detection antibody (1:99) was added and incubated for 60 min at 37 °C, followed by washing. HRP conjugate working solution (100 μL, 1:99) was added and plates incubated for 30 min at 37 °C. Plates were washed and 90 μL of substrate added to each well and incubated for 15 min at 37 °C, after which 50 μL stop solution was added and absorbance-read at 450 nm using the Victor Nivo^®^ multimode plate reader (Perkin-Elmer, Midrand, South Africa).

### 2.11. ERK1/2 (MAPK) Nuclear Translocation

Immunofluorescence (IF) microscopy was used to determine the nuclear translocation of ERK1/2 (MAPK). Cells were cultured on a sterile coverslip in 3.4 cm diameter culture plates. After irradiation and incubation, the cells were fixed (4% paraformaldehyde, 15 min at room temperature), washed (0.1% Tween-20 in PBS), permeabilised (0.1% Triton X-100 in PBS, 15 min at room temperature), washed, and blocked (1% BSA in PBS, 1 h at room temperature). Cells were exposed to unlabelled (1:250) primary antibody (Anti-MAP Kinase, activated (Dephosphorylated ERK-1&2) antibody, mouse monoclonal; M9692, Sigma-Aldrich, Johannesburg, South Africa) and incubated for 1 h at room temperature. The slides were then washed, and fluorescently labelled secondary antibody diluted in PBS-T (1:100) (anti-mouse, IgG, H&L, FITC conjugate; 12-506, Sigma-Aldrich, Johannesburg, South Africa) was added and incubated for 1 h at room temperature in the dark. Thereafter, the slides were washed and nuclei counterstained with 1 µg/mL 4′,6′-diamidino-2-phenelendole (DAPI), mounted, and observed under a fluorescent microscope, Carl Zeiss Axio Z1 (Carl Zeiss, Randburg, South Africa). Images were analysed by using ZEN 3.1 (ZEN pro) software (Carl Zeiss, Randburg, South Africa).

### 2.12. Statistical Analysis

Experiments were repeated 3 times (n = 3). Data are expressed as the mean ± standard error (SE) and each assay repeat was conducted in duplicate, the average of which was used. Experimental samples were compared to their non-irradiated controls (0 J/cm^2^). Student’s *t*-test was used to compare groups and One-Way Analysis of Variance (ANOVA) followed by Dunnett’s test to compare the differences between groups. Significant probability was accepted at * *p* < 0.05, ** *p* < 0.01 and *** *p* < 0.001. Cohen’s d was used to calculate the effect size for independent samples. Cohen’s d suggests that the effect size of less than 0.2 is considered to be a small effect size, 0.5 is considered to be a medium size, while larger than 0.8 is considered to be a large effect size.

## 3. Results

### 3.1. Cellular Morphology and Migration

Cellular morphology (all models) and migration (wounded models) was observed at 0, 24, and 48 h after irradiation at 660 nm with 5 J/cm^2^. Fibroblast cells showed normal characteristics and remained confluent throughout the experiment. In wounded cell models, the central scratch produced a cell-free zone that represented a “wound”, and the morphological changes were determined by the directional cell motility of the cells, probably in response to chemotaxis and the surface-compelled gradient of extracellular matrix (ECM).

**Figure 1:**
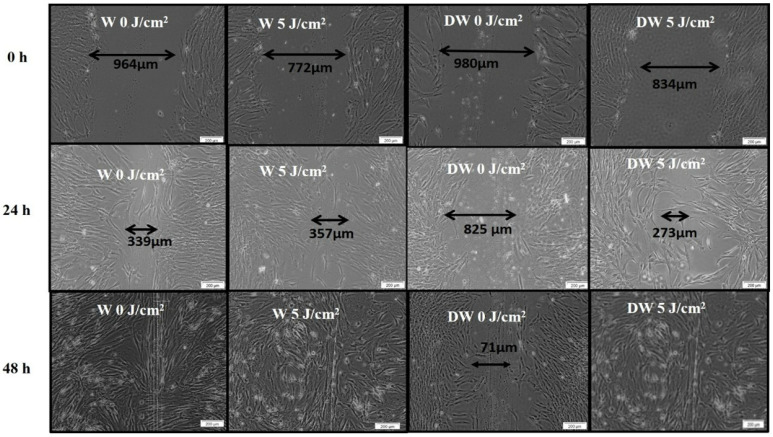
Micrographs depicting cell migration towards the centre of the central scratch as assessed by inverted microscopy at 0 h, 24 h, and 48 h following irradiation at a wavelength of 660 nm, with 5 J/cm^2^ in non-irradiated and irradiated wounded (W) and diabetic wounded (DW) cells.

**Figure 2:**
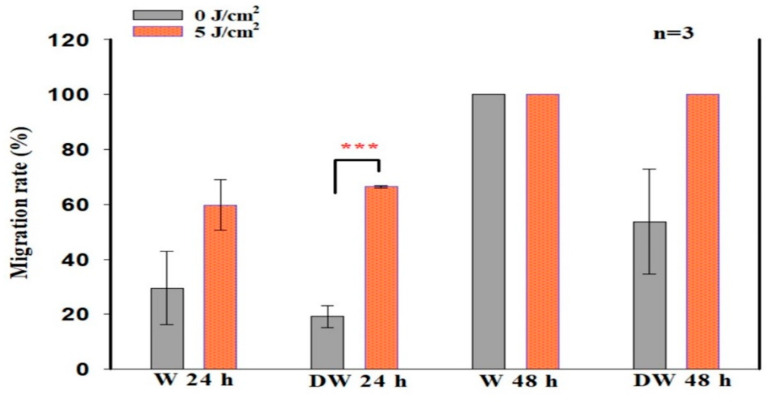
Migration rate (%) in non-irradiated (0 J/cm^2^) and irradiated (5 J/cm^2^) wounded (W) and diabetic wounded (DW) cell models, 24 and 48 h following irradiation at a wavelength of 660 nm. Data are expressed as ±SEM (n = 3). Significant probabilities compared to the non-irradiated controls are shown as *** *p* ≤ 0.001.

There was an increased presence of cells at the borders of the central scratch at 24 and 48 h post-irradiation. The central scratch in models that received irradiation was partially or completely closed by new cells, demonstrating the impact of PBM on both W and DW cells in vitro (Figure 1). The migration rate in non-irradiated (0 J/cm^2^) and irradiated (5 J/cm^2^) W and DW cells was calculated at 24 and 48 h following irradiation. The distance between the borders of the “wound” was assessed and the measurements (Table 2) used to determine the rate of migration in percentage (Figure 2). Irradiated DW cell models showed a significant increase in migration rate at 24 h post-irradiation (*p* < 0.001) with an effect size (d_Cohen_) of 2.0. There was complete wound closure at 48 h in non-irradiated and irradiated W cells, hence 100% migration rate, as well as in irradiated DW cells. The increased migration rate observed in irradiated DW cells at 48 h was not significant (*p =* 0.072).

### 3.2. Cellular Viability

**Figure 3:**
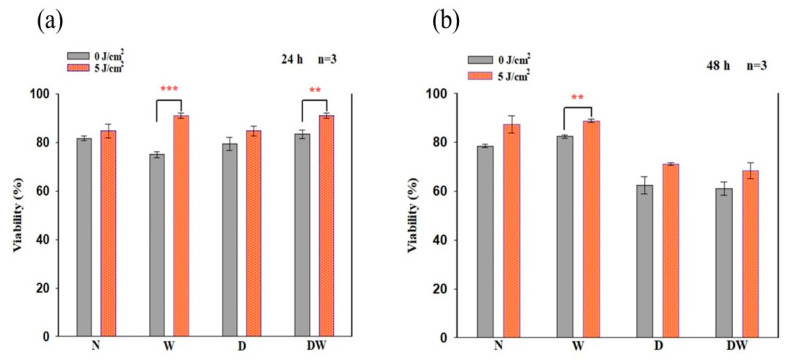
Viability of cells in percentage (%) as determined by the Trypan blue exclusion assay in non-irradiated and irradiated normal (N), wounded (W), diabetic (D), and diabetic wounded (DW) cells observed at 24 h (**a**) and 48 h (**b**) following irradiation at a wavelength of 660 nm with a fluence of 5 J/cm^2^. Data are expressed as ±SEM (n = 3). Significant probabilities compared to the non-irradiated controls are shown as ** *p* < 0.01 and *** *p* < 0.001.

In this study, cellular viability was determined using the Trypan blue exclusion assay at 24 and 48 h following irradiation (Figure 3). There was a significant increase in cellular viability in irradiated W cell models at both 24 and 48 h (*p =* 0.001 and *p* = 0.01, respectively) with an effect size (d_Cohen_) of 7.5 and 1.8, respectively. This increase was also seen at 24 h in DW cell models (*p* < 0.01; d_Cohen_ 3.5).

Comparison of non-irradiated N cell models to both irradiated and non-irradiated W, D, and DW cell models (one-way ANOVA) showed a significant decrease in cellular viability in non-irradiated W cell models (*p* = 0.05) and an increase in irradiated W cell models (*p* = 0.05) at 24 h (Figure 3a); at 48 h, there was an increase in irradiated W cell models (*p* = 0.001) (Figure 3b). When both non-irradiated and irradiated D cell models were compared to the same non-irradiated N cell models at 48 h, there was a significant decline in viability (*p* = 0.011 and *p* = 0.001, respectively). As compared to non-irradiated N cell models, there was a significant increase in viability in irradiated DW cell models at 24 h (*p* = 0.01), while at 48 h, there was a significant decrease observed in both non-irradiated and irradiated DW cell models (*p* < 0.001 and *p* = 0.003, respectively).

When comparing the wounded models, a significant increase was observed in cell viability at 24 h in non-irradiated DW cell models compared to non-irradiated W cell models (*p* = 0.001), while at 48 h, non-irradiated DW cell models showed a decrease (*p* = 0.001). There was a significant increase between irradiated W and irradiated DW cell models at 24 h (*p* = 0.003) and a significant decrease between irradiated W and irradiated DW cell models (*p* = 0.003) at 48 h.

### 3.3. Cellular Proliferation

**Figure 4:**
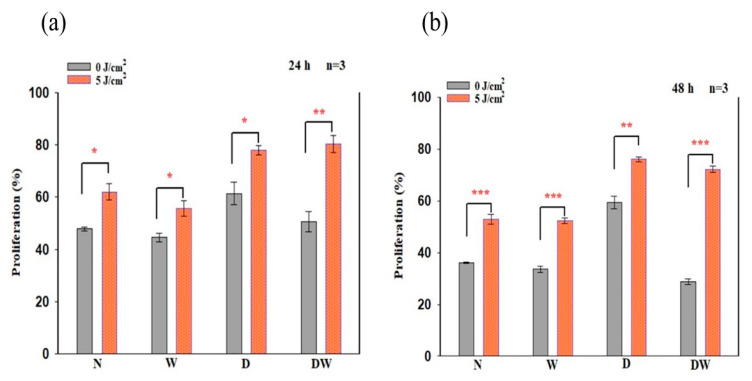
BrdU (5-bromo-2’-deoxyuridine) incorporation (% cellular proliferation) in non-irradiated and irradiated normal (N), wounded (W), diabetic (D), and diabetic wounded (DW) cell models following irradiation at 660 nm, with 5 J/cm^2^ at 24 h (**a**) and 48 h (**b**) determined by flow cytometry. Data are expressed as ±SEM (n = 3). Significant probabilities compared to the non-irradiated controls are shown as * *p* < 0.05, ** *p* < 0.01 and *** *p* < 0.001.

In this study, BrdU incorporation into dividing cells as evaluated by flow cytometry was used to quantify cellular proliferation. At 24 and 48 h following irradiation, a significant increase in cellular proliferation was observed in all the cell models (Figure 4), with effect sizes (dCohen) of 4.8 (N), 2.9 (W), 2.7 (D), and 6.0 (DW) at 24 h, and 7.6 (N), 7.6 (W), 5.8 (D), and 18.7 (DW) at 48 h, respectively.

Comparison of non-irradiated N cell models to non-irradiated W, D, and DW cell models (one-way ANOVA) at 24 h (Figure 4a) showed no significant changes. When non-irradiated N cell models were compared to irradiated W, D, and DW cell models at 24 h, irradiated D cells showed a significant increase in cellular proliferation (*p* = 0.001), while W and DW showed an insignificant increase (*p* = 0.066 and *p* = 0.100, respectively). Comparison of non-irradiated N cell models to non-irradiated W, D, and DW cell models (one-way ANOVA) at 48 h (Figure 4b) showed a significant increase in proliferation in non-irradiated D cell models (*p* = 0.001) and a decrease in DW cell models (*p* = 0.003). When compared to irradiated W, D, and DW cell models, a significant increase in proliferation was observed in irradiated D and DW cells (*p* = 0.001).

There was a significant decrease in cell proliferation at 48 h in non-irradiated DW cell models (*p* = 0.047) as compared to non-irradiated W cell models. In contrast, irradiated DW cell models showed a significant increase in cell proliferation at both 24 and 48 h (*p* = 0.001 and *p* = 0.006, respectively) compared to irradiated W cell models.

### 3.4. Basic Fibroblast Growth Factor (bFGF)

**Figure 5:**
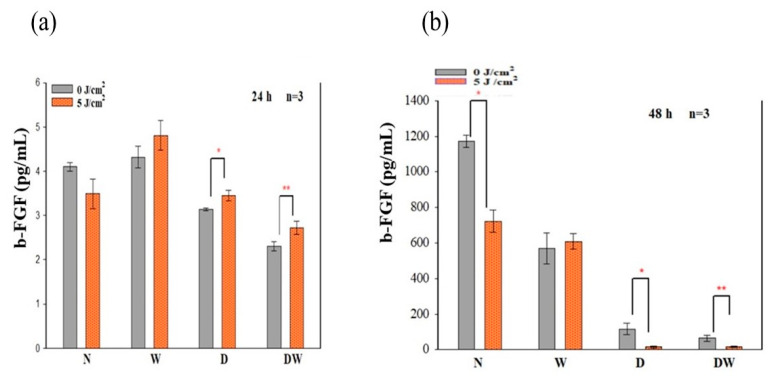
Concentration **of** bFGF release in media observed in non-irradiated and irradiated normal (N), wounded (W), diabetic (D), and diabetic wounded (DW) cell models at 24 h (**a**) and 48 h (**b**) following irradiation at 660 nm with a fluence of 5 J/cm^2^. Data are expressed as ±SEM (n = 3). Significant probabilities compared to the non-irradiated controls are shown as * *p* < 0.05, and ** *p* < 0.01.

BFGF/bFGF2 released from cells into the surrounding media was measured by ELISA at 24 and 48 h following irradiation (Figure 5). There was a significant increase in bFGF/FGF2 in irradiated D and DW cell models (*p* < 0.05 and *p* < 0.01, respectively) at 24 h, with an effect size (dCohen) of 4.6 and 6.8, respectively (Figure 5a); while at 48 h, a significant reduction was observed in N (*p* = 0.016; dCohen 6.7), D (*p* = 0.038; dCohen 4.6), and DW (*p* = 0.043; dCohen 6.8) cell models (Figure 5b).

Using one-way ANOVA, comparison of non-irradiated N cell models to the other non-irradiated and irradiated cell models (W, D, and DW) showed no significant changes in bFGF at 24 h (a). When non-irradiated N cell models were compared to non-irradiated and irradiated W, D, and DW cell models at 48 h (b), a significant decrease in bFGF/FGF2 was observed in non-irradiated and irradiated W (*p* = 0.003 and *p* = 0.007, respectively), D (*p* = 0.001 and *p* = 0.022, respectively), and DW (*p* = 0.001 and *p* = 0.007, respectively) cell models. When comparing the wounded models at 24 h, non-irradiated and irradiated W cell models showed increased bFGF compared to non-irradiated and irradiated DW cell models (*p* < 0.001), respectively. The same results were observed at 48 h (*p* < 0.001).

### 3.5. Phosphorylated Fibroblast Growth Factor Receptor (p-FGFR)

**Figure 6:**
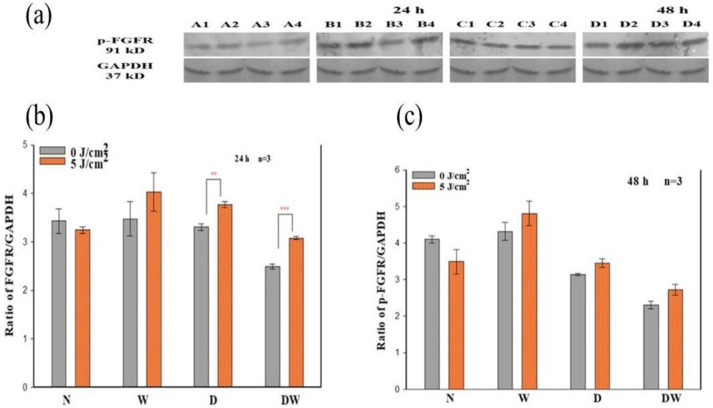
Effect of photobiomodulation on phosphorylated (p-) fibroblast growth factor receptor (FGFR) in normal (N), wounded (W), diabetic (D), and diabetic wounded (DW) cells irradiated at 660 nm with 5 J/cm^2^. GAPDH was used as a loading control. (**a**) Representative blots, A1: normal (0 J/cm^2^, 24 h); A2: wounded (0 J/cm^2^, 24 h); A3: diabetic (0 J/cm^2^, 24 h); A4: diabetic wounded (0 J/cm^2^, 24 h); B1: normal (5 J/cm^2^, 24 h); B2: wounded (5 J/cm^2^, 24 h); B3: diabetic (5 J/cm*^2^*, 24 h); B4: diabetic wounded (5 J/cm^2^, 24 h); C1: normal (0 J/cm^2^, 48 h); C2: wounded (0 J/cm^2^, 48 h); C3: diabetic (0 J/cm^2^, 48 h); C4: diabetic wounded (0 J/cm^2^, 48 h); D1: normal (5 J/cm^2^, 48 h); D2: wounded (5 J/cm^2^, 48 h); D3: diabetic (5 J/cm^2^, 48 h); and D4: diabetic wounded (5 J/cm^2^, 48 h). Quantification of the ratio of the intensity of protein-FGFR divided by GAPDH 24 h (**b**) and 48 h (**c**) post-irradiation. Data are expressed as ±SEM (n = 3). Significant probabilities compared to the non-irradiated controls are shown as ** *p ≤* 0.01, and *** *p* ≤ 0.001.

Determination of phosphorylated/activated FGFR was performed by Western blot. The band density ratio for p-FGFR was assessed by dividing it by the loading control (GAPDH) 24 and 48 h post-irradiation (Figure 6). Positive bands for p-FGFR were detected in non-irradiated and irradiated N, W, D, and DW cell models at both 24 and 48 h post-irradiation (Figure 6a). There was a significant increase in p-FGFR in irradiated D (*p* < 0.01; dCohen 2.1) and DW (*p* < 0.001; dCohen 1.7) cell models when compared to their control cell models 24 h post-irradiation (Figure 6b). However, at 48 h post-irradiation, there was no observable significant increase in any of the cell models (Figure 6c).

One-way ANOVA was used to evaluate differences in p-FGFR in cell models compared to non-irradiated N cells. A significant decrease was seen in non-irradiated DW cell models (*p* = 0.023) 24 h post-irradiation (Figure 6b). At 48 h, there was a significant decrease in both non-irradiated and irradiated D cells (*p* = 0.001 and *p* = 0.013, respectively), as well as non-irradiated and irradiated DW cells (*p* = 0.001) when compared to non-irradiated N cell models (Figure 6c). When comparing the wounded models at 24 h, no significant difference was observed in both non-irradiated and irradiated DW cell models when compared to both non-irradiated and irradiated W cell models, respectively. At 48 h post-irradiation, there was a significant increase in irradiated W cell models *p* = (0.050) and a significant decrease in non-irradiated DW cell models (*p* = 0.028) when a comparison was made to irradiated N cell models.

### 3.6. Phosphorylated (p)Ras

**Figure 7:**
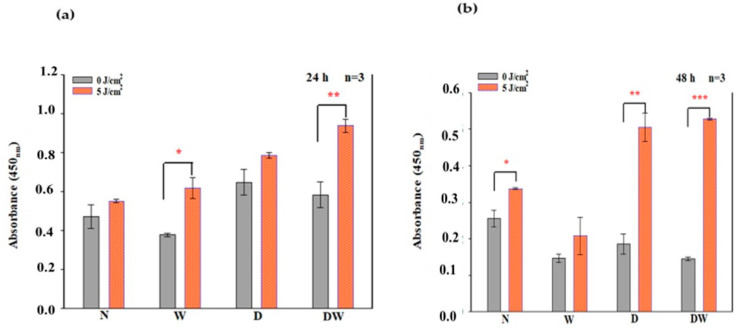
Phosphorylation (p-) of Ras-GFR1 as determined by ELISA in non- irradiated and irradiated normal (N), wounded (W), diabetic (D) and diabetic wounded (DW) cell models at 24 h (**a**) and 48 h (**b**) post-irradiation at 660 nm with a fluence of 5 J/cm^2^. Data are expressed as ±SEM (n = 3). Significant probabilities compared to the non-irradiated controls are shown as * *p* < 0.05, ** *p* < 0.01 and *** *p* < 0.001.

ELISA was used to analyse the activated/phosphorylated (p-) form of Ras at 24 and 48 h post-irradiation (Figure 7). At 24 h, irradiated W (*p* = 0.011; dCohen 5.3) and DW (*p* = 0.008; dCohen 4.1) cell models showed a significant increase in p-Ras (Figure 7a). At 48 h, a significant increase was observed in N (*p* ≤ 0.05; dCohen 4.1), D (*p* = 0.003; dCohen 6.6) and DW cells (*p* = 0.001; dCohen 6.5) (Figure 7b).

Using one-way ANOVA, comparison of non-irradiated N cell models to the other non-irradiated and irradiated cell models (W, D and DW) showed a significant increase in p-Ras at 24 h in irradiated D and DW cell models (*p* = 0.008 and *p* < 0.001, respectively) (Figure 7a). At 48 h post-irradiation, there was a significant decrease in non-irradiated W and DW cell models (*p* = 0.012 and *p* = 0.009, respectively), and a significant increase in irradiated D and DW cell models (*p* = 0.005 and *p* = 0.001, respectively) as compared to non-irradiated N cell models (Figure 7b).

When comparing the non-irradiated W models at 24 h to non- irradiated DW cell models, there was a significant difference noted in non-irradiated DW cell models (*p* = 0.04); however, at 48 h post-irradiation, there was no significant difference observed between both non-irradiated and irradiated W and DW cell models respectively (*p* = 0.921 and *p* = 0.957, respectively). When comparing the wounded models at 24 h, no significant difference was noted between both non-irradiated and irradiated DW cell models with non-irradiated and irradiated W cell models, respectively.

### 3.7. Phosphorylated (p-) MEK1/2

**Figure 8:**
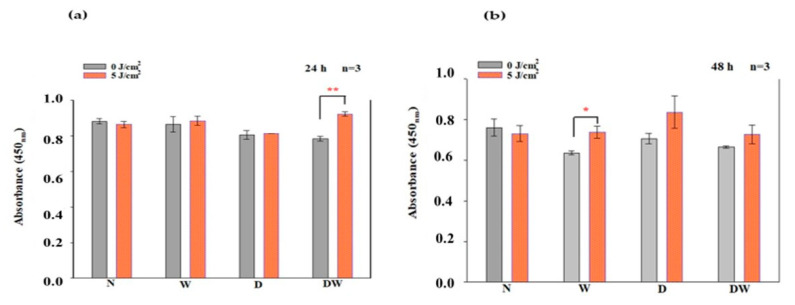
Phosphorylated (p-) MEK1/2 following irradiation at 660 nm and a fluence of 5 J/cm^2^, as determined by ELISA in non-irradiated and irradiated normal (N), wounded (W), diabetic (D), and diabetic wounded (DW) cell models at 24 h (**a**) and 48 h (**b**) post-irradiation. Data are expressed as ±SEM (n = 3). Significant probabilities compared to the non-irradiated controls are shown as * *p* < 0.05 and ** *p* < 0.01.

The in-cell ELISA was used to analyse activated/phosphorylated (p-) MEK1/2 at 24 and 48 h post-irradiation (Figure 8). At 24 h, irradiated DW cell models exhibited a significant increase in p-MEK1/2 (*p* = 0.002; dCohen 5.0) (Figure 8a). At 48 h, a significant increase was noted in W cell models (*p* = 0.03; dCohen 5.1) (Figure 8b).

Using one-way ANOVA, comparison of non-irradiated N cell models to the other non-irradiated and irradiated cell models (W, D, and DW) showed a significant decrease in non-irradiated DW cell models (*p* = 0.024) at 24 h post-irradiation (Figure 8a). At 48 h post-irradiation, there was no significant difference observed when cell models were compared to non-irradiated N cell models (Figure 8b). There was no significant difference observed in both non-irradiated and irradiated DW cell models when compared to non-irradiated and irradiated W cell models, respectively, at both 24 and 48 h.

### 3.8. Phosphorylated (p-) ERK1/2 (MAPK)

**Figure 9:**
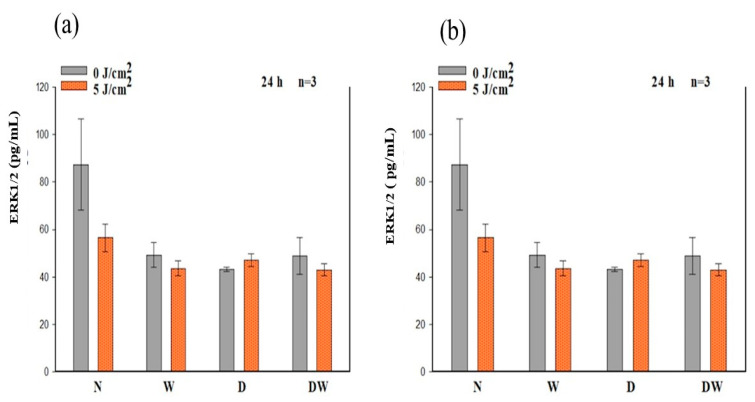
Phosphorylated (p-) ERK1/2 (MAPK) following irradiation at 660 nm and a fluence of 5 J/cm^2^, as determined by ELISA in non-irradiated and irradiated normal (N), wounded (W), diabetic (D), and diabetic wounded (DW) cell models at 24 h (**a**) and 48 h (**b**). Data are expressed as ±SEM (n = 3).

The in-cell ELISA was used to analyse activated/phosphorylated (p-) ERK1/2 24 and 48 h post-irradiation (Figure 9). There was no significant increase in (p-) ERK1/2 observed in all cell models when compared to non-irradiated control cells at 24 and 48 h. There was an increase in irradiated DW cells as compared to their control cells at 48 h, but the increase was not significant (*p* = 0.084).

Using one-way ANOVA, a significant decrease in p-ERK1/2 was observed in non-irradiated D cell models at 24 h as compared to non-irradiated N cell models (*p* = 0.030) (Figure 9a). At 48 h, no significant difference was observed in both non-irradiated and irradiated W, D, and DW cell models as compared to non-irradiated N cell models (Figure 9b). When comparing the wounded models, there was no significant difference observed in both non-irradiated and irradiated DW cell models compared to non-irradiated and irradiated W cell models, respectively, at 24 and 48 h.

### 3.9. Immunofluorescence

**Figure 10:**
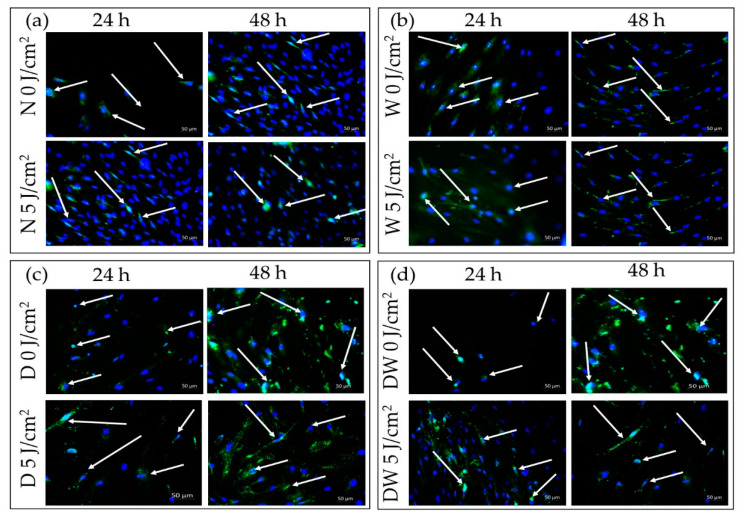
Immunofluorescence images of phosphorylated (p-) ERK1/2 (MAPK) in non-irradiated and irradiated normal (N) (**a**), wounded (W) (**b**), diabetic (D) (**c**), and diabetic wounded (DW) (**d**) cells at 24 and 48 h after irradiation at 660 nm with 5 J/cm^2^. Phosphorylated protein appears green (FITC; green signals), while the nuclei appear blue (DAPI; blue signal); 200× magnification.

In this study, immunofluorescence (IF) was conducted to visualize nuclear translocation of MAPK in non-irradiated and irradiated N, W, D, and DW cell models, 24 and 48 h after irradiation (Figure 10). Both non-irradiated and irradiated N, W, D, and DW cell models showed p-ERK signalling at 24 and 48 h following irradiation. Strong nuclear translocation was observed in irradiated DW cells as compared to their controls (non-irradiated cell models, 0 J/cm^2^) at 24 h (Figure 10d) and very little nuclear translocation in irradiated cells, with few localisations around the nucleus at 48 h following irradiation.

## 4. Discussion

This study aimed to determine if PBM at 660 nm and a fluence of 5 J/cm^2^ stimulates the Ras/MAPK signalling pathway via FGF binding and, in doing so, leads to increased cell proliferation and migration, ultimately leading to wound healing. PBM is a painless, non-invasive treatment to promote physiological changes and render therapeutic benefits. PBM has been shown to reduce inflammation and oedema, reduce pain, and promote wound healing [4,19,20], and in wound healing, it has been shown to increase cellular viability, proliferation, migration, and differentiation, as well as to cause vasodilation and increase collagen synthesis and growth factor release [16,21,22,23,24,25]. Many studies on PBM have used wavelengths that range from 630 to 980 nm and fluences between 0.1 and 100 J/cm^2^. These wavelengths penetrate the tissues with relative ease [26]. Although most PBM studies on wound healing make use of red and NIR light, a recent study showed that PBM with blue light (400–430 nm, 120 mW/cm^2^, 7.2 J/cm^2^) had a positive effect on chronic diabetic ulcers [27]. Blue light has been demonstrated to be beneficial for treating acute and chronic skin wounds, reducing inflammation, and promoting tissue regeneration [27]. Other studies have also reported that blue light holds some promise, particularly in the fight against excessive scarring and wound infections; however, it is crucial to consider the potential toxic and antiproliferative effects and to carefully consider the benefits and risks of using blue light to minimise the disturbances of skin physiology, which could result in impaired wound healing/closure and decreased scar breaking strength [28]. In this present study, a wavelength of 660 nm and a fluence of 5 J/cm^2^ was used. This fluence was selected as previous studies by the research centre have shown it to be an optimal fluence with this wavelength in the cell models used [13,16,21].

Biologically, for wound reduction and wound healing to take place, cellular migration must occur. In the present study, PBM at 660 nm and a fluence of 5 J/cm^2^ significantly increased cellular migration rate, with a large effect on irradiated DW cell models at 24 h (d_Cohen_ 2.0). A 20% migration rate was observed in non-irradiated control DW cell models, while a 70% migration rate was observed post-PBM. Complete closure was observed in non-irradiated and irradiated W cell models as well as irradiated DW cell models at 48 h, while non-irradiated DW cell models still showed some gaps, with an average wound distance of 142 μm between wound margins (migration rate of 68%). Incomplete wound closure in non-irradiated DW cells is expected, since under hyperglycaemic conditions, fibroblast migration is impaired. This debilitating effect was overcome by PBM, as shown by the significant increase in migration rate at 24 h and the complete wound closure at 48 h. The findings of the current study correlate with previous similar studies by Jere and colleagues [13] and Ayuk and colleagues [16] that showed that PBM at a wavelength of 660 nm and a fluence of 5 J/cm^2^ was able to stimulate cellular proliferation and migration [13,16]. Cellular migration is a key process in wound healing; therefore, for proper and timely cellular migration towards the injured tissue site and wound healing, the cells must be healthy and viable and capable of proliferating. In the present study, PBM had a large effect on cellular viability, as observed in irradiated W (24 and 48 h) and DW (24 h) cell models. Similarly, PBM had a large effect on all the cell models at both 24 and 48 h, including DW cell models. The result of the present study agrees with the study conducted by Ruhbar et al. [29], whereby PBM at a wavelength of 632 nm (power output 5 mW, power density 2.618 mW/cm^2^, irradiation time 192 s, and fluence 0.5 J/cm^2^) improved cellular proliferation and viability.

PBM has been shown to increase growth factor production [23,30]. bFGF facilitates wound closure through activation of growth, migration, differentiation, and survival of different cell types, including vascular endothelial cells and fibroblast cells [31]. In the present study, bFGF was significantly reduced in cell models which were grown under hyperglycaemic conditions (D and DW) as compared to cells grown under normoglycemic conditions (N and W). This demonstrates how hyperglycaemia affects bFGF release, further confirming the suitability of the diabetic cell model. When the diabetic models received PBM at 660 nm and a fluence of 5 J/cm^2^, a significant release of bFGF into the culture media was observed at 24 h and the Cohen’s d test showed that the effect of PBM on these cells was large. At 48 h post-PBM, a significant reduction in bFGF in irradiated D and DW cell models was observed. The researchers suggest that this decrease could be due to the consumption of bFGF by the cells in a paracrine fashion. Other studies have also found an increase in bFGF post-PBM. Khoo et al. [30] showed a significant increase in bFGF in diabetic fibroblast cells that received PBM (810 nm with 1 J/cm^2^). Castro et al. [32] and Damante et al. [33] showed an increase in FGF in non-diabetic human fibroblast cells irradiated at a wavelength of 780 nm and a fluence of 3–5 J/cm^2^, and in a wounded rat animal model irradiated at a wavelength of 660 nm and a fluence of 2–4 J/cm^2^, respectively. It is suggested that the biostimulatory effect of PBM at 660 nm and a fluence of 5 J/cm^2^ observed in the present DW cell models is likely due to the increased production and release of bFGF.

The presence of DM weakens the functional status of growth factor receptor signalling and the downstream protein activation that results in decreased cellular activities [34]. In the present study, PBM (660 nm and 5 J/cm^2^) stimulated the release of bFGF and associated activation of FGFR and associated downstream proteins. The results showed that PBM had a large effect on p-FGFR, with significant increases seen in D and DW cell models 24 h post-PBM. These results are in agreement with the significant increase in bFGF observed in the same models. The increase in the activated receptor at 48 h was no longer evident, which corresponds with the decrease observed in bFGF. Non-irradiated DW cell models (control) showed a significant decrease in p-FGFR (and a corresponding decrease in bFGF) when compared to non-irradiated W cells, again showing the effect of hyperglycaemia on these cells. There are no studies available on the effect of PBM on the activation of FGFR. A few studies have found activation of other growth factor receptors or upregulation of genes for these receptors in response to PBM. A study done by Illescas-Montes et al. [35] found a significant increase in the gene expression of TGF-β as well as the receptors TGF-βR1 and TGF-βR2 in fibroblast cells after a single or double (72 h between irradiations) treatment with PBM using a 940 nm diode laser (0.5 mW, 4 J/cm^2^). A study similar to the present study conducted by Jere and colleagues [13], whereby fibroblast DW cell models were irradiated at a wavelength of 660 nm with a fluence of 5 J/cm^2^, found a significant increase in EGF and p-EGFR 48 h following irradiation.

Ras, MEK1/2, and ERK1/2 (MAPK) are all stimulated when FGFR is activated. ERK1/2 then translocates into the nucleus and activates transcriptional genes that promote cell migration and proliferation [36]. Ras signalling has been found to be a key regulator of wound healing. This was shown in a study whereby aspirate from chronic venous leg ulcers demonstrated decreased FGF, and cell proliferation was obstructed through defects in cell-cycle progression advancement from the G1 phase to the S phase. This inhibition was discovered to be via a Ras-mediated signalling pathway involving MAPK [37]. Sepe and colleagues [38] demonstrated that directed cell movement is critical for wound closure in an in vitro wound healing experiment, and Ras is closely involved in controlling this movement and wound closure. In this study, phosphorylation of Ras, MEK1/2, and ERK1/2 (MAPK) was determined. When DW cells were irradiated with a diode laser at 660 nm with 5 J/cm^2^, there was a large effect and significant increase in p-Ras and p-MEK1/2 at 24 h. At 48 h, this effect on p-Ras was still evident. This increase in p-Ras and p-MEK1/2 correlates with the increase in bFGF and p-FGFR in the DW cell models at 24 h. There were no significant changes observed in p-ERK1/2 (MAPK). Irradiated DW cell models showed a rise in p-ERK1/2 (MAPK) after 48 h; however, the increase was not significant and is possibly due to the large variation and standard deviation observed in the control cells. Immunofluorescence images showed nuclear translocation of p-ERK1/2 (MAPK). Studies have shown that PBM regulates the activation of Src, Ras, and MAPK [38]. Ye et al. [39] show that PBM at 1064 nm enhanced collagen synthesis while inhibiting degradation via activation of ERK1/2 and JNK/MAPK in a rat model. The findings of this study also relate to those of Ejiri et al. [40], who conducted an in vitro investigation and irradiated human gingival epithelial cells to high frequency (30 kHz) PBM at a wavelength of 904–910 nm. PBM increased cell proliferation and migration, as well as activation of ERK/MAPK, enhancing gingival wound healing [40].

This study had some limitations, which include the absence of additional cells that might be present at the wound site and the lack of cellular interactions with fibroblasts. Additionally, immunofluorescence images of ERK1/2 (MAPK) nuclear translocation were not quantified.

## 5. Conclusions

The findings of this study have demonstrated that PBM at 660 nm with 5 J/cm^2^ activated the Ras/MAPK signalling pathway in diabetic wounded cells in vitro through the increased release and binding of bFGF to FGFR. This led to increased cellular migration, viability, and proliferation. It is established that these pathways are dysregulated under hyperglycaemic conditions, and PBM has been shown to reverse these effects. There might be several other signalling pathways that were activated via the same route; therefore, more research is needed to confirm the PBM-related molecular mechanisms in both wounded and diabetic wounded cells, as well as to characterise specific signalling pathways that are activated to provide a better understanding of PBMs role in diabetic wound healing. PBM at 660 nm and 5 J/cm*^2^* has been demonstrated to be effective in stimulating diabetic ‘wounded’ cells in vitro. Although these results cannot be directly translated to the in vivo situation, it does provide strong evidence of mechanisms of action of PBM using light in the red spectrum, especially since the study looked at the activated forms of the signalling proteins.

## Figures and Tables

**Table 1:** Laser parameters.

Variables	Diode Laser
Wavelength (nm)	660
Fluence (J/cm^2^)	5
Power density (mW/cm^2^)	11
Power output (mW)	100
Spot size (cm^2^)	9.1
Energy (J)	47.7
Irradiation time (s)	454
Emission	Continuous

**Table 2:** Average distance of three repeats of the wound length in µm in wounded (W) and diabetic wounded (DW) cell models (n = 3). Significant probability related to non-irradiated (0 J/cm^2^) control cells at the same time-point is shown as * *p* ≤ 0.05 (SEM). Significant probability related to 0 h within cell models is shown as † *p* ≤ 0.05 and †† *p* ≤ 0.01.

	W		DW	
	0 J/cm^2^	5 J/cm^2^	0 J/cm^2^	5 J/cm^2^
0 h	448	558	592	415
24 h	272 †	230 *	457	159
48 h	0 †	0	142	0 ††

## Data Availability

Data available on request.
